# (+)-Catechin & Proanthocyanidin Fraction of *Uncaria gambir* Roxb. Improve Adipocytes Differentiation & Glucose Uptake of 3T3-L1 Cells Via Sirtuin-1, Peroxisome Proliferator-Activated Receptor γ (PPAR γ), Glucose Transporter Type 4 (GLUT-4) Expressions

**DOI:** 10.34172/apb.2020.072

**Published:** 2020-08-09

**Authors:** Silvy Arundita, Friardi Ismed, Rauza Sukma Rita, Deddi Prima Putra

**Affiliations:** ^1^Department of Biochemistry, Faculty of Medicine, Andalas University, Limau Manis, Padang, 25163, West Sumatera, Indonesia.; ^2^Faculty of Pharmacy, Andalas University, Limau Manis, Padang, 25163, West Sumatera, Indonesia.

**Keywords:** Diabetes mellitus, Obesity, Proanthocyanidin, Sirtuin-1, *Uncaria gambir* Roxb., 3T3-L1

## Abstract

***Purpose:*** To improve adipocytes differentiation & glucose uptake activity of 3T3-L1 cells through sirtuin-1, peroxisome proliferator-activated receptor γ (PPAR γ), glucose transporter type 4 (GLUT-4) of (+)-catechin & proanthocyanidin fraction *Uncaria gambir* Roxb.

***Methods:*** Adipocytes differentiation activity of (+)-Catechin of* Uncaria gambir* Roxb. was determined by oil red O staining method & glucose uptake activity was determined by measuring 2-deoxyglucose uptake on 3T3-L1 cells. The ability of (+) - catechin as an activator of sirtuin-1 was assessed by administration of (+) - catechin with the presence of a specific inhibitor of sirtuin-1, nicotinamide. Metformin 1 mM & 5 mM were used as positive control. Sirtuin-1, PPAR γ & GLUT-4 expressions were determined by RT-PCR.

***Results:*** (+)-Catechin & proanthocyanidin fraction of *Uncaria gambir* Roxb. were found to increase adipocyte differentiation & glucose uptake by increasing activity of sirtuin-1 as well as metformin (*P* ≤0.05). PPAR γ, GLUT-4 and sirtuin-1 expressions were known to be responsible for this activities.

***Conclusion:*** These results indicate that (+)–catechin & proanthocyanidin fraction of *Uncaria gambir* Roxb. could be utilized as a renewable bioresource to develop potential antidiabetic and antiobesity agents.

## Introduction

Diabetes mellitus patients in 2000 recorded as many as 171 cases and was estimated to be 366 million in 2030. Diabetes mellitus may result in decreased body productivity due to complications caused. Obesity is a physiological condition which is a major risk factor for type 2 diabetes mellitus.^1^ There were previous studies suggested natural compounds containing polyphenols have a positive impact on the development of type 2 diabetes mellitus related to obesity.^2^ *Uncaria gambir* Roxb. is one of the plants found in Southeast Asia that is used by traditional communities to treat diabetes because of its polyphenol compounds.^3^

The main polyphenol in *Uncaria gambir* Roxb. are the catechin family with the major compound is (+) -catechin. Catechins on *Uncaria gambir* Roxb. has two forms, monomers and polymers. Catechin polymer which is found in the dimer forms is proanthocyanidin. Proanthocyanidin is a dimeric compound of catechins which is more larger & polar than catechins.^4,5^ Procyanidin B3,was known to have an activity as an alpha glucosidase inhibitors which were stronger compared to (+)-catechin and acarbose was used as a positive control. Procyanidin B3 had an IC_50_ value of 17.3 µM, (+)-catechin 53.8 µM, and acarbose 312.6 µM.^5^

The cell which is responsible for the development of obesity are adipocyte cells. Adipocyte cells in addition to have a function in storage, it also has an ability to secrete various proteins protein in the form of enzymes, transcription factors and carrier molecules.^5,6^ Proteins such as sirtuin-1, peroxisome proliferator-activated receptor γ (PPAR γ), and GLUT-4 have an important role in the development of obesity and type 2 diabetes mellitus.^7^ Sirtuin-1 is a master regulator enzyme found in adipocyte cells and plays a role in the body’s energy balance. PPAR γ is a transcription factor that is responsible for the differentiation of adipocyte cells. Glucose transporter type 4 (GLUT-4) is a carrier molecule that is responsible for glucose uptake activity to enter adipocyte cells.^6,7^ Sirtuin-1 had been hypothesized to play a role in the regulation of PPAR γ and GLUT-4 genes. There were several studies showing adipose cells in obese patients expressing expression of sirtuin-1 and PPAR γ in small amounts.^3,8^ Polyphenol compounds such as catechins & its derivatives were proven to stimulate the activity of sirtuin-1, PPAR γ, & GLUT-4.^2,3,8^

The research on (+)-catechins & proanthocyanidins as an antidiabetic from *Uncaria gambir* Roxb. is still at the Cellular level and has not explained further about its molecular mechanism. This research will investigate the effect of (+)-catechins and proanthocyanidin fraction of *Uncaria gambir* Roxb. on differentiation and glucose uptake activity through sirtuin-1, PPAR γ, GLUT-4 in 3T3-L1 adipocyte cell.

## Materials and Methods

### Chemicals 

(+)–Catechin was from *Uncaria gambir* Roxb. The purity of (+)–catechin was approximately 99.9 % and were purchased from PT. Andalas Sitawa Fitolab, Padang, Indonesia (Code: RC-03401). Mouse 3T3-L1 fibroblast (CL-173) was kindly given by Prof. M. Taher. from International Islamic University Malaysia (IIUM). Glucose uptake kit was Promega Glucose Uptake-Glo™ J1342 (Madison, Wisconsin, USA). TRIzol reagent was purchased from Invitrogen (Waltham, Massachusetts, USA). cDNA Synthesis Kit was purchased from Bioline BIO-65054 (Tauton, MA, USA). cDNA was amplified using PCR Bioline SensiFAST SYBR No-ROX kit BIO-98005 (Tauton, MA, USA). All other chemicals were analytical grade and purchased from Sigma Aldrich, Singapore. The period of study was from February 2017 to September 2019.

### Plant material and Preparation of proanthocyanidin fraction

Five kilograms fresh leaves of *Uncaria gambir* Roxb. harvested in Kebun Tumbuhan Obat Farmasi (KTOF) UPT, Sumatran Biota Laboratory, Andalas University. The sample specimen was identified and deposited in the herbarium of Andalas University (No: 343/K-ID/ANDA/2019). The leaves were laid at oven (50 ^o^C, 24 h). All of the sample were pulvered by a grinder.

The powdered leaves of *Uncaria gambir* Roxb., were then soaked in methanol 70% for 9 days at room temperature with frequent agitation for a period of 3 days. The methanol extract was subsequently filtered through Whatman No. 1 filter paper. Further step, to promote the rapid removal of excess solvent, the samples were put on flask of rotary evaporator to obtained extraction yields 18.65%.

Liquid-liquid extraction was done against 500 g of methanol extracts with hexane, ethyl acetate, and butanol to obtained hexane fraction (0%), ethyl acetate fraction (40.6%), butanol fraction (10%). 10 g of butanol fraction was directly subjected to a Diaion HP-20 (Sigma Aldrich, Singapore) column (9 cm i.d. x 60 cm) with H_2_O by increasing amounts of MeOH in stepwise gradient elution to obtain 10 subfractions SA01–SA10, respectively. SA03 was a proanthocyanidin fraction, a brown powder 31.2 mg (0.32%). A brown bottle storage was required for all of the Diaion HP-20 fractions until further use.

### HPLC analysis

**Figure 1:**
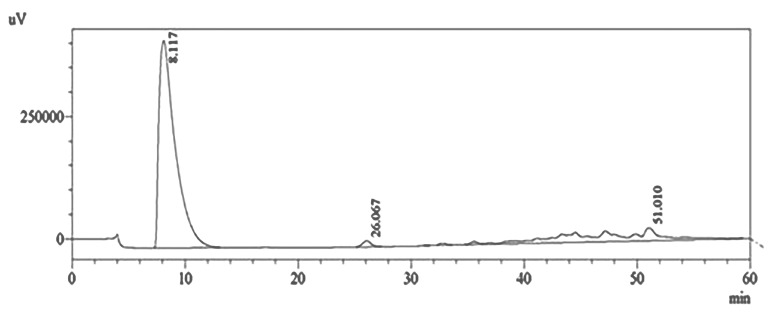
The purity of proanthocyanidin fraction was determined using HPLC analysis. The amount was measured by HPLC technique. The peak proanthocyanidin fraction was observed at retention time of 8117 min.

HPLC system (Shimadzu, Kyoto, Japan) was carried out with a binary gradient pump (LC-10AD) at ambient temperature (25-28°C) using the Hypersil BSD C18 column (4.6 × 100 mm, size 3 μm) (Thermo Scientific, USA) with the C18 guard column. Water (A) and methanol (B) as a mobile phase were sent at a flow rate of 1000 mL/min based on programmed gradient elution: 100% (A) isocratic for 5 minutes, 90% (A) for 5 minutes, 80% (A) during 5 minutes, 70% (A) for 5 minutes, 60% (A) for 5 minutes, 50% (A) for 5 minutes, 40% (A) for 5 minutes, 30% (A) for 5 minutes, 15% (A) for 5 minutes, 5 % (A) for 5 minutes, 0% (A) isocratic. The total running time was 60 minutes. All mobile phase solutions were made on the day of implementation. The quantification of the proanthocyanidin fraction was set at 254 nm. The results were analyzed using ChemStation software version 6 (Figure 1).

### Proanthocyanidin content

Proanthocyanidin content in the SA03 fraction was determined by the vanillin-HCl assay described by Sun et al. ^9^ To 100 μL of study solution or methanol (as a control) in a study tube was added with 2.5 mL of 8% hydrogen chloride in glacial acetic acid and 2.4 mL of 1% vanillin in glacial acetic acid solution. The reaction mixture was homogenized and incubated for 5 minutes. The absorbance at 510 nm was measured using Spectrophotometer UV-VIS 3600 (Shimadzu, Japan). The data for proanthocyanidin contents were expressed as mg of (+)-catechin equivalent weight (CAE)/100 g of dry mass (% b/b).

### Cell culture 

Cultures of 3T3-L1 cells were performed as described. Confluent preadipocytes were treated withSupplement in a cocktail of 10% fetal bovine serum (FBS) and 1% combined antibiotic penicillin-streptomycin (10 000 μg/mL) added on 37 C incubator in a 5% humidified CO_2_ atmosphere in Dulbecco’s modified Eagle’s medium (DMEM).

### Adipocyte differentiation

The cells were grown on 6 well-plates with a density of 1 × 10^5^ cells/well. Two days later (defined as day-0), the cells were driven for differentiation using a differentiating medium i.e DMEM contained 10% FBS & MDI [0.5 mM 3-isobutyl-1-methylxanthine (IBMX), dexamethasone 0.25 deM, and 1 mg/mL of insulin] for 2 days. In order to determine the adipocyte differentiation activity, 3T3-L1 preadipocytes were given various concentrations of study compounds (10.75; 21.5; 43 μM of (+)-catechin & 25, 50, 100 μg/mL of proanthocyanidin fraction in differentiating media (starting day 0). On the second day, media differentiation was replaced with 10% FBS media in DMEM added 1 μg/mL of insulin for two days (4th day). The cell was then maintained at 10% FBS in DMEM for the next 4 days (day 8) with media changes every 2 to 3 days.^10^ Metformin 1 and 5 mM were used as positive controls. Adipocytes differentiation scheme was summarized in Table 1.

**Table 1:** Differentiation cocktails of adipocyte

**Day**	**Negative Control**	**Positive Control**	**Treatmen Group**
0	DMSO 0,01 %, Medium (DMEM/ 10% FBS), MDI	DMEM, 10% FBS, & MDI (IBMX 0.5 mM, dexamethasone 0.25 µM & insulin 1 μg/mL), metformin 1 and 5 mM	DMEM, 10% FBS, & MDI (IBMX 0,5 mM, dexamethasone 0.25 µM and insulin 1 μg/mL), (+)catechin or proanthocyanidin fraction
2	DMEM/ 10% FBS and insulin 1 μg/mL	DMEM, 10% FBS and insulin 1 μg/mL	DMEM, 10% FBS and insulin 1 μg/mL
4-8	DMEM/ 10% FBS	DMEM/ 10% FBS	DMEM/ 10% FBS

### Viability study

This study was used to assess whether the sample inhibits population viability. Viability study was performed by 3- (4,5-dimethyltiazol-2-il) -2,5-diphenyltetrazolium bromide (MTT) according to Mosman.^11^ 2 ×10^4^ of 3T3-L1 cells are grown in 96 well plates and differentiation process had been completed. Subsequently, 86, 43, 21.5 μM (+) –catechin & 100, 50, and 25 μg/mL proanthocyanidin fraction were incubated for 24 hours.^10,12^ 20 μL of MTT stock solution (5 mg/mL) was added to each well & incubation was carried out during 4 hours at 37°C for the next 100 𝜇L DMSO was added. Read the absorbance at 570 nm on the Promega Glomax microplate reader SA3030 (Madison, Wisconsin, USA). The concentration of the sample that caused the decrease in cell viability compared to the negative control was not used in further studies.

### Oil red O staining study

This study aims to determine the effect of (+)–catechins and proanthocyanidin fraction *Uncaria gambir* Roxb. on the cell differentiation activity of 3T3-L1 cell. Eight days after the differentiation process, cells were washed three times with PBS and allowed to stand in the presence of 4% formalin for 15 min at room temperature. After the formalin period, cells were washed with PBS and given an Oil Red O dye solution (3 parts of 0.5% Oil Red O in isopropanol and 2 parts of water) for 30 minutes.^10^ Cells were washed twice with water, dried thoroughly and visualization were placed under an Olympus microscope (Tokyo, Japan). Quantitative analysis was carried out by dissolving cells and oil red with isopropanol, a general oil solvent and optical density of 520 nm were performed by a Promega Glomax microplate reader SA3030 (Madison, Wisconsin, USA).

### Deoxyglucose uptake study

Uptake glucose activity was analyzed by measuring 2-deoxyglucose (2DG) uptake by 3T3-L1 cells according to the manufacturer’s instruction. A mature adipocyte 3T3-L1 cells were incubated for 1 hours with (+)-catechin 10.75; 21.5; 43 µM, proanthocyanidin fraction 25, 50, & 100 µg/mL, and 1 mM metformin in PBS. The luminescence signal was detected as a relative light unit (RLU) on the Promega luminometer SA3030 (Madison, Wiscosin, USA).

In order to investigate the inhibitory activity of 2DG uptake through sirtuin-1 on adipocytes, mature adipocytes 3T3-L1 were treated with sirtuin-1 specific inhibitors, nicotinamide. The nicotinamide 30 mM was used in this assay based on preliminary study.Nicotinamide was added 30 min before addition of (+)-catechin, proanthocyanidin fraction, and metformin.^13^ Calculation of 2DG6P concentration was based on the 2DG6P standard curve.

### A quantitative reverse transcriptase polymerase chain reaction (qRT-PCR) study

RNA from adipocyte cell were obtained using TRIzol reagent. The purity of RNA was known by A260/A280 ratio using Thermo Scientific NanoDro spectrophotometer 2000 (Waltham, MA, USA). The obtained mRNA was then transcribed backwards to cDNA using the SensiFAST cDNA Synthesis Kit according to the manufacturer’s instructions. cDNA was amplified using PCR SensiFAST SYBR No-ROX kit on RT-PCR devices (BioRad CFX96). The mRNA oligonucleotide sequences obtained using Geneious Prime (version 2020.0.4) were as follows: mouse GLUT-4 (F: 5-CAG CTC TCA GGC ATC AAT -3 & R : 5-TCT ACT AAG AGC ACC GAG-3), PPAR γ (F: 5-CCA AGA ATA CCA AAG TGC GA-3 & R: 5-TGC TTT ATC CCC ACA GAC TC-3), sirtuin-1 (F: 5-GTT CGT GGA GAT ATT TTT AAT CAG-3 & R: 5-GGG TAT AGA ACT TGG AAT TAG TGC-3), & Beta-actin as a control (F:5-ACA CCC CAG CCA TGT ACG-3 & R: 5 TGG TGG TGA AGC TGT AGC C-3. The results were presented in the form of mRNA expression levels relative to control.

### Statistics

Data were calculated as mean ± SD from three experiments. ANOVA SPPS version 19 was used. If the data were normally distributed, the post hoc study employed was Tukey HSD andif the data were not normally distributed, a Kruskal-Wallis was performed followed by Mann-Whitney. A *P* ≤ 0.05 was considered as significant.

## Result and Discussion

### Identification of proanthocyanidin fraction

The purity of proanthocyanidin fraction of SA03 from *Uncaria gambir* Roxb. which was identified by HPLC methods (Figure 1) and proanthocyanidin content was > 75% eq. (+)–catechin as identified by the vanillin-HCl assay.^14^

### Study of (+)-catechin &proanthocyanidin fraction on the viability of 3T3-L1 cells

Viability study was a study to determine the number of living cells due to variations in the treatment of (+)-catechin and proanthocyanidin fraction of *Uncaria gambir* Roxb. on the 3T3-L1 cells. The highest concentrations of (+)-catechin 43 𝜇M and proanthocyanidin fraction 200 µg/mL both of them had no effect on the cell viability (*P* > 0.05, Data were normally distributed). (+)-Catechins had a viability inhibiting activity of 50% at a concentration of 9.24 mM, while the proanthocyanidin fraction was 239.88 µg/mL.

Other researchers indicated the same result with our study. The effect of (-)-epigallocatechin gallate, a family of catechins, at a dose of >50 μM showed a decrease of viability on 3T3-L1 cell.^15^ The statement of Sakurai et al in 2009 was also in accordance with this study. (+)–catechin at a highest dose of 43 µM, still showed high viability that was not significantly different with control (*P* > 0.05).^16^

The results of XTT (sodium 30-[1-[(phenylamino)-carbonyl]-3,4-tetrazolium] -bis (4-methoxy-6-nitro) benzenesulfonic hydrate assay showed that 300 μg/mL proanthocyanidin-rich extracts 68.4% did not show a change in viability of 3T3-L1 cells.^17^ The dose of the proanthocyanidin fraction 50 μg/mL, in this study, was the only dose that did not change viability. A dose of 100 μg/mL caused a decrease in viability of 24% and a dose of 200 μg/mL by 40%. This was slightly different from previous studies. The reason for these difference was a variation of a plant sources, parts, extraction & isolation methods, as well as the viability study methods employed. Previous researcher used the *Pinus maritima* bark while in our study we used leaves from the *Uncaria gambir* Roxb. differences in the origin of a plant may cause differences in the compounds contained therein.^2,5^

### Differentiation activity assay by (+)-catechin and proanthocyanidin fraction Uncaria gambir Roxb 

**Figure 2:**
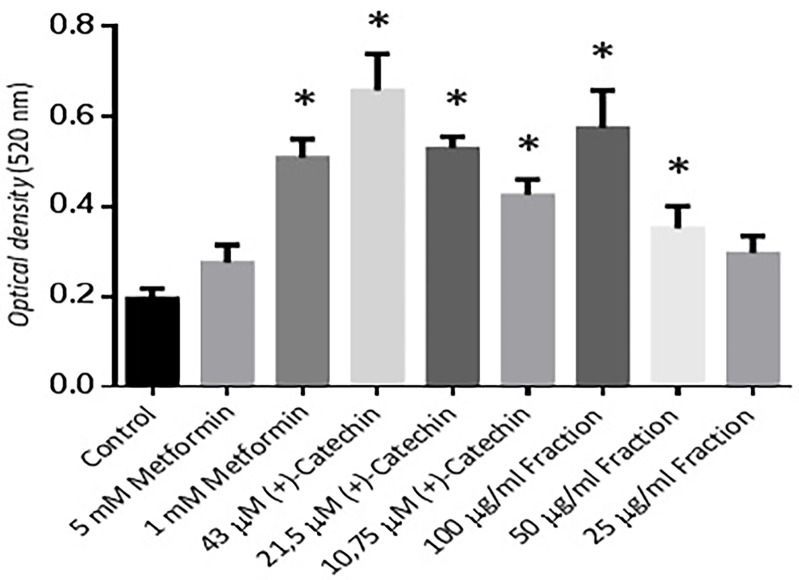
Effect of different concentration of (+)-catechin & proanthocyanidin fraction on the differentiation of 3T3-L1 adipocytes. Data is represented as mean ± SD, with n = 3 per group. *P ≤ 0.05 compared to control.

Differentiation activity assay was done using Oil red O staining. Non-toxic concentrations based on MTT viability study for (+)-catechins were 43, 21.5, & 10.75 μM and proanthocyanidin fraction were 100, 50, 25 μg/mL. The effect of the positive control groups and the treatment groups compared to the negative control in this study were showed to be significantly (*P* ≤ 0.05) except for the proanthocyanidin fraction 25 μg/mL which was not significantly different (*P* > 0.05) (Figure 2).

Metformin 1 and 5 mM in this study proved to have an ability to increase 3T3-L1 cell differentiation compared to negative control. This result was consistent with previous research which stated that a low-dose of 1.25-2.5 mM metformin stimulated cell differentiation by 1.7 times compared to negative control.^18^ The previous study mentioned that 5-10 mM metformin had a lower differentiation activity than metformin 1 mM.

12.5, 25, 50, 100, and 200 μM (+)-catechins of *Rhododendron groenlandicum* (Oeder) Kron & Judd (Labrador tea) stimulated 3T3-L1 cell differentiation measured by triglyceride accumulation of 1.1-2.5 times compared to control.^19^ The results of this study were in agreement with our research. (+)–Catechins in our research increased cell differentiation activity. A 10.75 to 43 µM (+)–catechin increased cell differentiation by 2-3 times compared to negative control (*P* ≤ 0.05). The negative control group showed significantly different from the 100 and 50 µg/mL proanthocyanidin fractions (*P* ≤ 0.05). All data were normally distributed.

The previous study indicated that proanthocyanidin –rich extracts from grape seeds had an ability to reduced the size and increase the amount of rat adipose tissue.^20^ Polyphenols in their activation against AMPK, interestingly, had been reported to be stronger than metformin by 50 to 200 times.^21,22^ These findings caused (+)-catechins, a polyphenol compound, had a stronger action or almost the same as metformin. An increase in AMPK activity would lead to an increase in the activity of the sirtuin-1 enzyme & PPAR γ, two genes who responsible for the activity of cell differentiation.^23,24^

The 50 and 25 µg/mL proanthocyanidin fraction, however, their cell differentiation activity was seen to be lower than metformin. This was probably caused by the structure of proanthocyanidin which has more polar groups than metformin.^5,25,26^ Other supporting studies revealed that flavonoids which have a hydroxyl groups do not exhibit an increasing effect of triglyceride amount & cell differentiation effects on adipocyte cells.^27,28^

### Glucose uptake study by (+)-catechin **&**proanthocyanidin fraction UncariagambirRoxb

This study was designed to evaluate the effect of (+)-catechin and proanthocyanidin fraction on the glucose uptake activity. The 2-deoxiglucose molecule is a molecule that has an ability to interact in the same way as glucose. It can interact with glucose transporters & can be phosphorylated by the hexokinase enzyme & becomes a 2DG6P.^29^ The activity of 2-deoxiglucose uptake was analyzed by measuring the concentration of 2DG6P that successfully formed on 3T3-L1 adipocyte cells.

**Figure 3:**
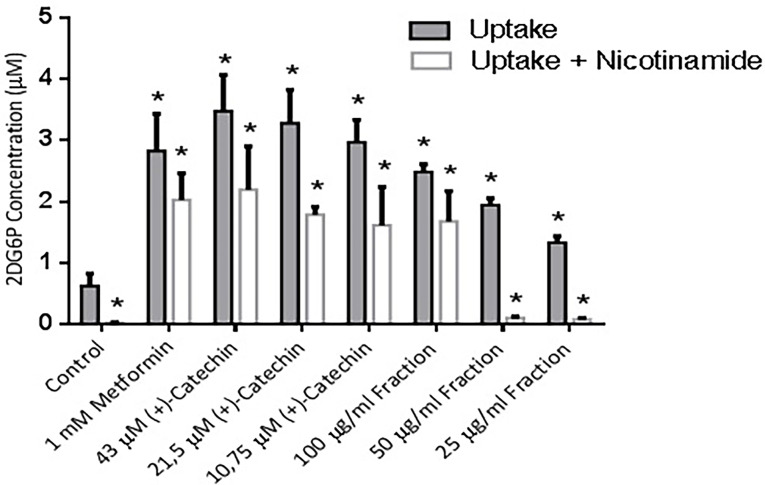
The effects of different concentration of (+)-catechin & proanthocyanidin fraction in glucose uptake of 3T3-L1 adipocytes. Data is represented as mean ± SD, with n = 3 per group. *P  ≤ 0.05 compared to control.

To determine the uptake activity of 2-deoxy-D-glucose (2DG), differentiated 3T3-L1 cell were treated with (+)-catechin & proanthocyanidin fractions for 1 hours. The cells were treated with 1 mM metformin as a positive controls. The 2DG uptake effect on 1 mM metformin, 43, 21.5, & 10.75 μM (+)-catechins & 100, 50, 25 μg/mL proanthocyanidin fractions compared to the negative control had been shown to be statistically significant based on Mann-Whitney study (*P* ≤ 0.05). 1 mM metformin increased 2DG6P concentration by 4 times compared to negative control. This result was in agreement with previous studies reporting 1 mM metformin increased 2-deoxy-D-[3H] glucose uptake by 4 times compared to negative control^10,30^ (Figure 3).

The 50 µM (-)–catechin in the presence of insulin was increased 2-deoxy-D-[14C] glucoseby 1.2 times compared to negative control of 3T3-L1 cells.^31^ A florescence based-method of 200 µg/mL proanthocyanidin-rich extract (purity: 68.4% ) from *Pinus maritima* stem bark increased 2DG uptake by 1.2 times compared to negative control.^18^ Proanthocyanidin extracts of grape seeds (140, 105, and 70 µg/mL) stimulated 2-deoxy-d-[^3^H]glucose uptake by 5, 2.6, and 2.2 times compared to negative control.^32^ All the data of (+)-catechins & proanthocyanidin fractions were vary due to the location of growing plant, sample extraction methods, and glucose uptake methods variation.

The (+)-catechins showed no significant difference of 2DG6P concentrations against 1 mM metformin (*P* > 0.5). Metfromin and polyphenols had been shown to increase glucose uptake activity through AMPK, sirtuin-1, and GLUT 4.^21,22,33^ The previous report was in line with our research which stated metformin and (+)-catechins had no significant difference of 2-deoxyglucose uptake activity (*P* > 0.05). Proanthocyanidin, a phenolic group compound, showed a slight difference of 2DG uptake activity from catechins. Proanthocyanidin structure is larger and has more polar property than metformin.^4,25^ It tends to cause a weaker effects in 2DG uptake activity of 3T3-L1 cell.^5,26^ Proanthocyanidin fractions exhibited a lower 2DG6P concentrations than (+)-catechin significantly (*P* ≤ 0.05). Chemical structures that are more lipophilic tend to show a higher glucose uptake activity and GLUT-4 translocation in adipocyte cells, but not in muscle cells.^25,26^

Research on activator sirtuin-1 as an antidiabetic has received attention for a long time. This was because of the Sirtuin-1 gene decline in patients with type 2 diabetes mellitus.^34^ Sirtuin-1 plays a role in increasing activity of glucose uptake through the carrier molecule, GLUT-4, in adipocyte cells.^3,4,12^ Metformin, which was a well-established antidiabetic drug, showed an increase on AMPK enzyme activity. It resulted in a sirtuin-1 increasing activity.^35^ Polyphenol compound, resveratrol, increase glucose analog uptake activity and act as an sirtuin-1 activator.^12,36^

The effect of nicotinamide was statistically significant based on Mann-Whitney study which was characterized by the decrease of a glucose uptake activity compared to the groups without nicotinamide administration (*P* ≤ 0.05). The result was in line with the previous research conducted by Xiao et al.^33^ The administration of nicotinamide to all of the treatment groups, in this study, showed a significant difference in the decrease of 2DG6P compared to the treatment group without nicotinamide administration (*P* > 0.05).

### Role of (+)-catechin and proanthocyanidin fraction in PPAR γ, GLUT-4, and sirtuin-1 expression

**Figure 4:**
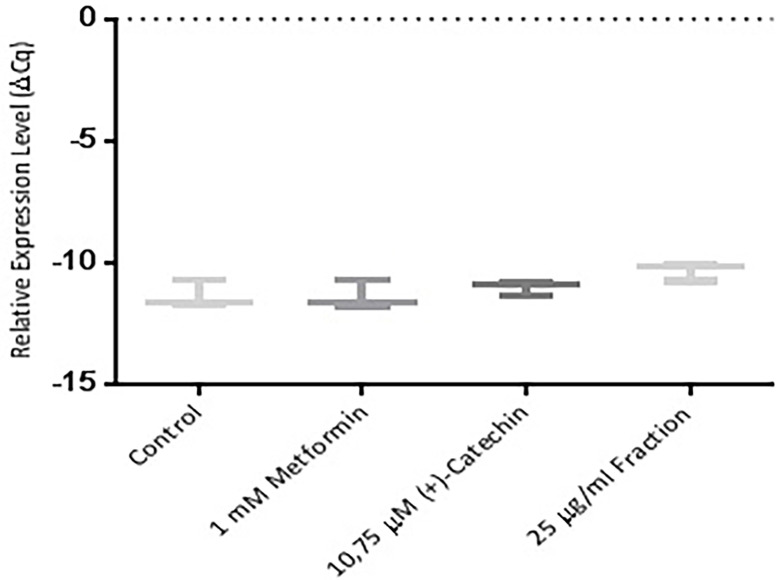
Effect of metformin 1 mM, (+)-catechin (10.75 μM), proanthocyanidin fraction (25 μg/mL) on sirtuin-1 expression. Data is represented as mean ± SD, with n = 3 per group. *P  ≤ 0.05 compared to control. Cq : Quantification level.

**Figure 5:**
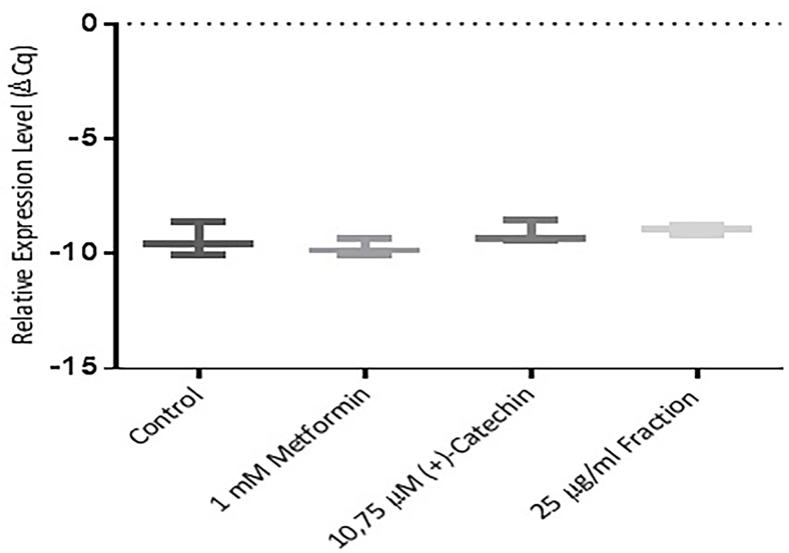
Effect of metformin 1 mM, (+)-catechin (10,75 μM), proanthocyanidin fraction (25 μg/mL) on PPAR γ expression. Data is represented as mean ± SD, with n= 3 per group. *P  ≤ 0.05 compared to control. Cq : Quantification level.

**Figure 6:**
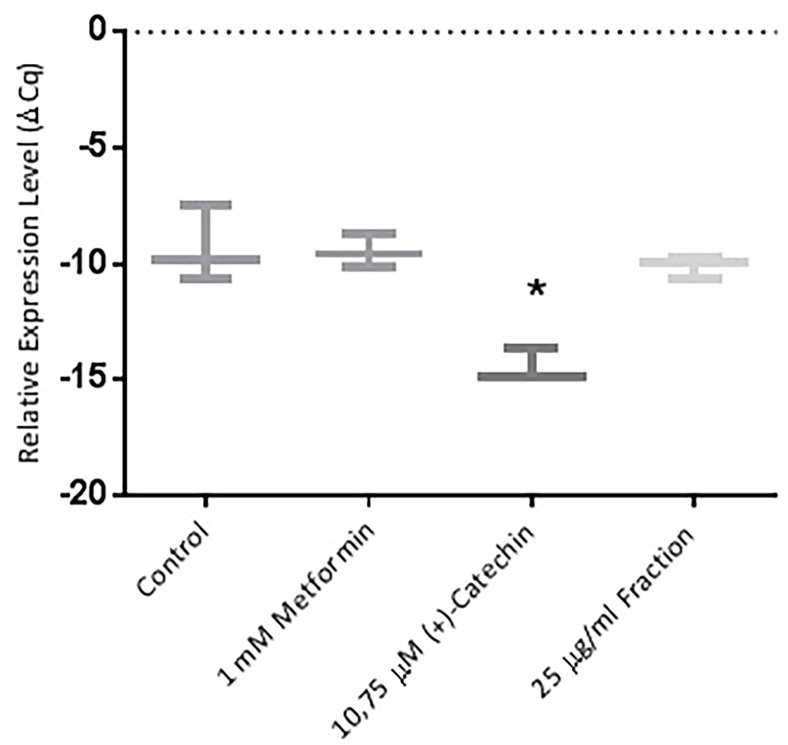
Effect of metformin 1 mM, (+)-catechin (10,75 μM), proanthocyanidin fraction (25 μg/mL) on GLUT-4 expression. Data is represented as mean ± SD, with n= 3 per group. *P  ≤ 0.05 compared to control. Cq : Quantification level.

Further study were an attempt using the RT-PCR method. This study was performed to find out whether (+)-catechin & proanthocyanidin fraction *Uncaria gambir* Roxb. induce cell differentiation and glucose uptake activity at mRNA levels. The results showed that (+)-catechin (10.75 µM) and proanthocyanidin fraction (25 μg/mL ) both could increase the excretion of sirtuin-1 mRNA (Figure 4), PPAR γ (Figure 5, and GLUT-4 (Figure 6). Metformin, (+)-catechin, and fraction of proanthocyanidin had been shown to increase sirtuin-1 expression compared with negative controls (*P* > 0.05, All the datas were normally distributed). This was in line with the previous study of which stated that metformin is an activator of sirtuin through the *in silico* study.^35^ Epigallocatechin gallate 10 μM increased 2DG6P uptake through sirtuin-1 by florescence method.^33^ The cell differentiation activity was regulated by PPAR γ gene.^7,8^ An increase in PPAR γ expression would cause an increase in the cell differentiation activity of insulin-sensitive adipose cells.^10^ Metformin, (+)-catechin, and the proanthocyanidin fraction were shown to increase PPAR γ expression compared to negative controls (*P* > 0.05). These results were in line with previous study which stated that 1 mM of metformin stimulated AMP-activated protein kinase (AMPK) through decreasing the regulation of phosphatase and tensin homolog (PTEN). PTEN inhibition improved insulin sensitivity. The improvement of insulin sensitivity was very closely related to the increased cell differentiation activity of adipocyte cells. Increased AMPK activity by metformin thus also had an impact on increasing PPAR γ gene.^37^ (+)-Catechins which increase PPAR γ gene were in accordance with the previous study. (-)-Catechins derived from green tea have been shown to induce adipocyte differentiation in human bone marrow mesenchymal stem cells (hBM-MSCs) through stimulation of PPAR γ transcription activity.^38^ There were studies, however, stated that (-)-epigallocatechin gallate, (-)-catechin 3-gallate and (-)-epigallocatechin suppressed the differentiation of adipocyte cells.^16,39^ This difference was due to variation in research methods. Catechins, on those studies, were given at the end of the differentiation stage (late stages, day 8), while this study, catechin was given at an early stage (day 0).^16,39^ Pinent et al in 2004 study was in accordance with this study. Proanthocyanidin from grape seed stimulated PPAR γ gene expression.^32^ Glucose uptake activity was regulated by the GLUT-4 gene.^7,10^ (+)-catechin (*P* ≤ 0.05). Metformin and proanthocyanidin fraction (*P* > 0.05) have been shown to increase PPAR γ expression compared to negative controls. This was in accordance with previous study, which stated that 1 mM Metformin increased PPAR γ gene expression compared to negative control.^10^ Ueda et al in 2010 declared that 50 μM non gallate type catechins increased GLUT-4 gene expression in 3T3L1 cells.^12^ Proanthocyanidin from elderberry extract increased GLUT-4 in muscle cells.^40^

The main regulatory enzyme, sirtuin-1, regulates cell differentiation & glucose uptake activity in adipocyte cells. Thus, an increase in the sirtuin-1 gene leading to the increase of PPAR γ and GLUT-4 gene.^7^ The study results at the mRNA level were in agreement with those shown in the cell differentiation and 2DG uptake activities. Increased levels of sirtuin-1, PPAR, and GLUT-4 mRNAs were manifested by an increase in cell differentiation and 2DG uptake activity.^6-8^

Mature adipocyte cells developed from the cell differentiation process of pre-adipocyte cells, which its presence is needed for controlling whole energy balance and glucose homeostasis. This is evidenced by research stating GLUT-4 gene is only expressed in adult fat cells.^41,42^ The limitations of the present study were the *in vivo* study had not yet been operated. For this reason, an *in vivo* study needs to be carried out for further research.

## Conclusion

In summary, this research brought a new evidence that (+)-catechin and proanthocyanidin fraction of *Uncaria gambir* Roxb. had an ability to increase the sirtuin-1 expression which was proven on the increase of cell differentiation activity which was confirmed via PPAR 𝛾 expression and glucose uptake via GLUT-4 expression. These results indicated that (+)-catechin & proanthocyanidin fraction of *Uncaria gambir* Roxb. may be a candidate for preventing obesity particularly among diabetic patients.

## Ethical Issues

Not applicable.

## Conflict of Interest

Authors declare no conflict of interest in this study.

## Acknowledgments

The authors wish to sincerely thank Prof. Dr. Muhammad Taher (International Islamic University Malaysia) who kindly provided the 3T3-L1 cells. This research work was supported by a grant from the Ministriy of Research, Technology, & Higher Education Republic of Indonesia, (Grant No.324/SP2H/LT/DRPM/IX/2016).
